# Assessing work capacity – reviewing the what and how of physicians’ clinical practice

**DOI:** 10.1186/s12875-020-01134-9

**Published:** 2020-04-27

**Authors:** P. Nordling, G. Priebe, C. Björkelund, G. Hensing

**Affiliations:** 1 School of Public Health and Community Medicine, The Sahlgrenska Academy at University of Gothenburg, Box 453, SE-405 30 Gothenburg, Sweden; 2 Region Västra Götaland, Närhälsan Research and Development Primary Health Care, Gothenburg, Sweden

**Keywords:** Work capacity evaluation, Physicians, Review, Work ability, Sick leave

## Abstract

**Background:**

Although a main task in the sickness certification process, physicians’ clinical practice when assessing work capacity has not been thoroughly described. Increased knowledge on the matter is needed to better understand and support the certification process. In this review, we aimed to synthesise existing qualitative evidence to provide a clearer description of the assessment of work capacity as practiced by physicians.

**Method:**

Seven electronic databases were searched systematically for qualitative studies examining what and how physicians do when they assess work capacity. Data was analysed and integrated using thematic synthesis.

**Results:**

Twelve articles were included. Results show that physicians seek to form a knowledge base including understanding the condition, the patient and the patient’s workplace. They consider both medical and non-medical aspects to affect work capacity. To acquire and process the information they use various skills, methods and resources. Medical competence is an important basis, but not enough. Time, trust, intuition and reasoning are also used to assess the patient’s claims and to translate the findings into a final assessment. The depth and focus of the information seeking and processing vary depending on several factors.

**Conclusion:**

The assessment of work capacity is a complex task where physicians rely on their non-medical skills to a higher degree than in ordinary clinical work. These skills are highly relevant but need to be complemented with access to appropriate resources such as understanding of the associations between health, work and social security, enough time in daily work for the assessment and ways to better understand the patient’s work place. Also, the notion of an “objective” evaluation is questioned, calling for a greater appreciation of the complexity of the assessment and the role of professional judgement.

## Background

Sickness absence is a concern for many countries as it is costly for both individuals and the society as a whole. For the individual, consequences such as loss of daily routines and work-related social network in combination with short- and long-term negative effects on income [1] can be problematic. For society, the financial costs are substantial and include loss of production at work places and sickness benefit expenditures. In Sweden, for example, the direct cost for sickness benefits was about 8 billion EUR in 2018 (approx. 2% of GDP) [2]. Consequently, through various reforms, many European countries have tried to reduce public finance expenditure on sickness benefits during the last decade.

In most European countries, physicians are key actors in the sickness certification process as they are responsible for the assessment and certification of symptoms, function, ability to work and need for social benefits and/or rehabilitation, thereby serving as gate-keepers towards the financial resources of society. As such, they are expected to manage this task satisfactorily, but concerns have been raised regarding their practices. A general assumption put forward regarding physicians’ sick-listing practices is that physicians are too liberal in sick-listing. They are described as relying too much on patients’ subjective reports, i.e. to value the doctor-patient relationship higher than upholding their gate-keeper function [3, 4] and patients are described as having a strong influence on sick-listing decisions [5, 6]. Other concerns refer to variation in how physicians assess and evaluate work ability. A vignette study showed e.g. that different insurance physicians’^1^ assessment of work ability of the same patient varied between 0 and 80 (0 = no work ability, 100 = full work ability) [7]. Similar findings are presented in a study of multidisciplinary medical expert teams where the team doctors assessed patients’ work ability higher than both the patients and their treating physicians [8].

In line with this, multiple studies have shown that from the physicians’ perspective sickness certification is intricate [9–14], even to the point where they wish for someone else to do the job [15]. Problems that have been identified are time constraints, lack of tools, insufficient education in insurance medicine, role conflicts and possible negative effects on the doctor-patient relationship [14, 16–19]. Also, the insurance system itself and/or (the lack of) collaboration with other stakeholders are presented as causing problems, such as difficulties completing certification forms and risk of breaching patient confidentiality when communicating with employers [17, 20, 21].

The assessment of work capacity is a central part in sickness certification as in many countries the disease itself does not legitimate social benefits but only reduced function due to the disease. Of all parts of the sickness certification process physicians seem to find the assessment of work capacity the most difficult one [18]. In a Swedish national evaluation of sickness certificates only 45% of certificates provided enough information to establish the right to sickness benefits. Most commonly information on ‘activity limitation’ was missing – a crucial piece of information when determining work capacity [22].

To our knowledge, previous research on physicians’ sickness certification practices has rarely focused on the assessment of work capacity. The purpose of this study was to evaluate and synthesize existing research, aiming to answer the pressing research question: “What do physicians do when they assess work capacity as part of the sickness certification process, and how do they do it?”. Increased knowledge on this matter is needed to better understand and support the certification process.

## Method

The methodology for qualitative reviews is debated [23], some proposing the same principles as for quantitative reviews while others finding the principles of qualitative primary research more suitable. The latter was applied in this review and it is presented in accordance with the ENTREQ checklist, see Additional file 1.

### Search process and quality assessment

**Fig. 1:**
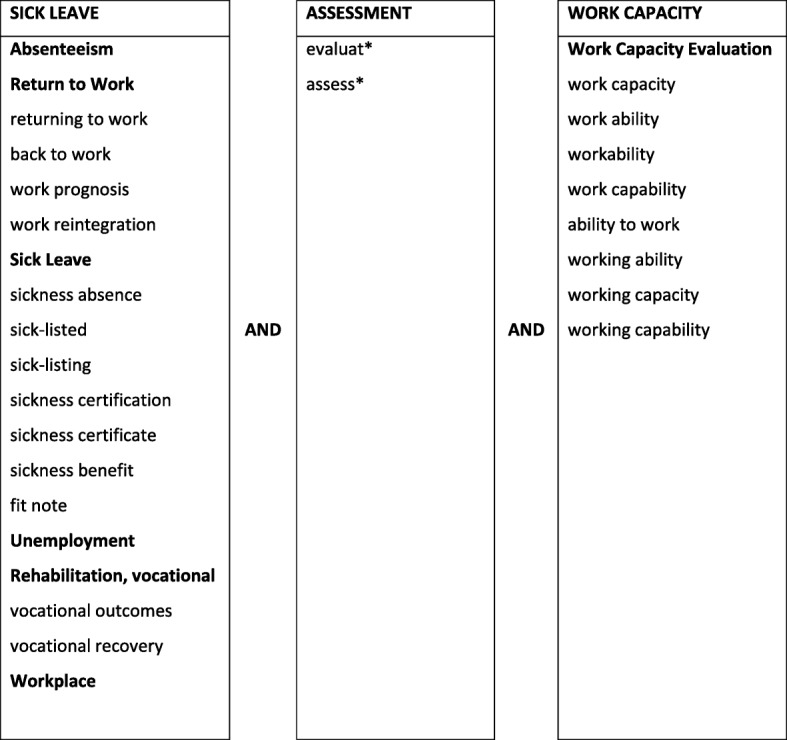
The search strategy. All keywords in the three domains were combined in the following way: (absenteeism OR return to work OR …) AND (assess* OR evaluat*) AND (work capacity evaluation OR work capacity OR …). MeSH-terms in bold. *With all possible endings

A systematic literature search was carried out in March 2016 in seven electronic databases covering the main scientific disciplines of sickness absence research: PubMed, CINAHL, Scopus, Web of Science, PsycInfo, Sociological abstracts and AMED (all years until April 2016, in English, peer-reviewed). The search strategy was developed in collaboration with information specialists at the Gothenburg university library at the medical faculty and contained keywords related to work capacity, assessment and sick leave (a proxy for work capacity), see Fig. 1. An updated search, using the same search strategy but for the time period January 2016 – September 2019, was carried out in August 2019. A manual search was done by going through reference lists of included articles.

The purpose was to identify and analyse the findings of qualitative studies describing physicians’ practices when assessing work capacity in the sick-listing process. Inclusion criteria were i) the study investigated physicians’ daily work with sickness absence matters, ii) the results discussed, fully or to a large extent, the assessment of work capacity and iii) the study included a qualitative research approach. We sought to understand how the assessment was carried out and understood by physicians, i.e. our focus was not on accounts of formalized procedures. Therefore, studies on permanent disability claims or specific instruments or methods were excluded, as they were interpreted as not reflecting physicians’ everyday work or own knowledge and handling of the matter. Consequently, we use the term “work capacity assessment” in a general way, referring to the physician’s own process of assessing of the patient’s ability to perform his/her work and the need for sick leave.

PN screened the abstracts. When reading the full-text papers, articles were assessed regarding relevance: PN assessed whether the content was in line with the aim and research question, PN and GH then jointly evaluated the richness of data. Articles that was not considered rich enough, i.e. that only discussed work capacity assessments briefly, were excluded. Included articles were assessed regarding quality by GH and CB independently, using the CASP checklist (see Additional file 2), with criteria for inclusion set to at least 5 of 6 “Yes” in part A and at least 2 of 4 “Yes” in part B and C.

### Data extraction

Our overall research question was “What do physicians do when they assess work capacity and how do they do it?” with specified research questions such as “what questions do they ask?” and “what resources do they use?”

First, a descriptive analysis was made to identify authors, country of origin, year of publication, aim, study design, year of data collection, method for analysis, informants (number and specialty) and sampling method. Then, the procedures of thematic synthesis were followed to combine the results of included studies and develop key themes that reflected results in the different studies [24]. Included articles were read in full several times to get an overview of the material. The “Results” sections of included articles were chosen as units of analysis. Only data that explicitly concerned physicians was analysed; in cases of uncertainty authors were contacted for clarification. The first author read and analysed all articles and compared the findings with the co-authors who each read and analysed a selection of articles. Interpretations and themes were continually discussed with the co-authors and any inconsistencies were resolved. Through reading the text line-by-line meaning units relevant to the research question were identified and coded inductively. When meaning units did not fit an existing code, a new code was created. Then, similar codes were grouped into sub-categories, which were then grouped into categories. Codes, sub-categories and categories were revised and reorganized until no new sub-categories or categories were identified. Finally, themes summarizing the essence of the categories were formulated. The analysis was done manually, no computer software was used.

## Results

**Fig. 2:**
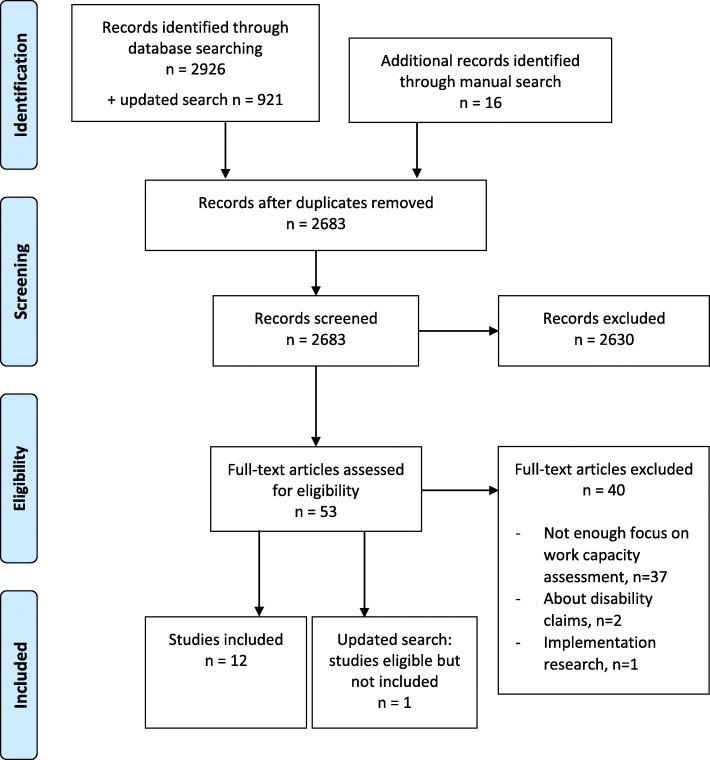
PRISMA Flow Diagram showing the results from the systematic search and selection of studies

The results of the literature search are presented in Fig. 2.

Twelve studies [25–36] were included in the analysis. All papers met the CASP quality criteria. The studies were performed in the years 2003–2011 (publication year 2007–2013) and almost exclusively in Europe. Five originated from Sweden, two from Norway, two from The Netherlands, one from the UK, one from Ireland and one from Canada. The majority were based on interviews, individual or focus group, but there was also one ethnographic study and two studies based on written text (open-ended answers to a questionnaire and written statements in sickness certificates respectively). Number of participants (only physicians counted) ranged from 6 to 62 (median 14; mean 19), 228 in total. In the study analysing sickness certificates, 475 certificates were included. A majority of physicians (59%) were general practitioners (GPs), about one third (35%) were insurance physicians (IPs) and the rest (6%) miscellaneous, e.g. orthopaedic surgeons.

In the updated search, one study was identified as meeting the inclusion and quality criteria [37]. As it was identified after completion of the analysis it was not included in the synthesis, but is commented on in the discussion section.

Characteristics of included studies are presented in Table 1.

**Table 1:** Characteristics of included studies

Authors Year of publication Country	Aim	Study design Year of data collection Method for analysis	Informants Sampling method	Main findings relating to our research question
Foley et al. [25] UK/Ireland	Explore the information seeking process in GPs’ fitness for work consultations.	Questionnaire with open-ended questions regarding vignettes presenting different hypothetical fitness for work consultations (physical or psychological complaint, +/− social problem and/or request for/reluctance to sick leave). 2011 Thematic analysis and content analysis	62 general practitioners (25 men, 37 women) Random sample	GPs seek different information depending on diagnosis.
Krohne & Brage [29] Norway	Examine GPs’ experiences of new rules regarding functional assessments in sickness certification.	Focus group discussions 2003–2004 Systematic text condensation	23 general practitioners (19 men, 4 women) Recruitment through a medical association, outside the influence of the authors.	The functional assessment was described by GPs as an implicit part of the medical examination which was difficult to describe. Lack of objective measures required trust in the patient’s story, which depended on several factors.
Krohne & Brage [26] Norway	Investigate how GPs conceptualize functioning in relation to sickness certification.	Focus group discussions 2003–2004 Systematic text condensation	23 general practitioners (19 men, 4 women) Recruitment through a medical association, outside the influence of the authors.	Functioning was understood as a complex construct including both physical, social and mental ability. However, in clinical practice, physical ability was emphasized.
Meershoek et al. [27] The Netherlands	Examine the ways physicians assess the eligibility of clients for sickness and disability benefits.	Ethnographic study with > 500 observations of consultations between physicians and patients. The physicians were also interviewed. - Inductive content analysis as in grounded theory	20 insurance physicians -	The assessment of eligibility for sickness benefits is a reasoning rather than a measurement. A medical diagnosis is insufficient for assessing work ability. Both medical and social aspects are taken into consideration.
Nilsing et al. [30] Sweden	Investigate what aspects, according to the ICF* model, physicians consider when assessing a patient’s functioning and work ability. *International Classification of Functioning, Disability and Health	Analysis of descriptions of the patient’s functioning in sickness certificates 2007 Mixed methods: Content analysis with ICF as a conceptual framework; various statistical analyses	475 sickness certificates Consecutive collection of all certificates in a new sick-leave period during 2 weeks.	Overall, functioning was described mostly as bodily impairments. Limitations in activity and participation were mentioned to a lesser extent and environmental factors almost not at all. Which aspects were considered was related to diagnosis and physician specialty/affiliation.
Nilsing et al. [28] Sweden	Explore primary health care professionals’ experiences of the sick leave process.	Semi-structured focus group discussions 2011 Qualitative content analysis	18 health care professionals in primary health care: 6 physicians, 3 physiotherapists, 4 occupational therapists and 5 counsellors Purposive sampling	Work capacity in conditions based on clinical findings was found easy to assess due to the physicians’ medical competence, while in subjective conditions it was described as either having trust in the patient’s story or as guessing. Lack of knowledge of work place factors added to the insecurity.
Slebus et al. [31] The Netherlands	Examine what aspects, according to the ICF* model, physicians consider when assessing work capacity *International Classification of Functioning, Disability and Health	Telephone interviews: participants answered three questions regarding work capacity assessment of a certain patient category (musculoskeletal, psychiatric or ‘other’). 2005 Content analysis with ICF as a conceptual framework	60 insurance physicians Random sample	The physicians predominantly considered aspects of body function and participation, while personal and environmental factors were not often mentioned. Different aspects were considered important depending on diagnosis.
Soklaridis et al. [32] Canada	Explore FPs views on handling work disability assessments, challenges when assessing work ability and ways to improve the process.	Semi-structured in-depth interviews - Descriptive phenomenologic approach	Six family physicians -	The work ability assessment was seen as a complex process where psychosocial factors need to be considered.
Stahl et al. [33] Sweden	Examine the relationship between professionals in Swedish interdisciplinary rehabilitation teams, focusing on the definitions and uses of the concept of work ability.	1. Semi-structured focus groups 2. Individual interviews 2006–2007 Qualitative content analysis	1. Twelve interdisciplinary teams at primary health care centres (PHCCs), 66 participants in total. The teams normally include physician, occupational therapist, physiotherapist, medical social worker and social insurance officer. 2. The twelve managers of the PHCCs where the interdisciplinary teams were located. Purposive sampling	The physicians have a holistic view on work capacity and include both medical and non-medical factors in their assessment. This view is not shared by the Social Insurance Agency (who decides on the right to benefits), which creates tension. Meeting the patient seldom and briefly, as well as lacking knowledge of the patient’s work place hampers the assessment. Collaboration with other health professionals (occupational therapists, physiotherapists) might improve the assessment.
Stigmar et al. [34] Sweden	Describe physicians’ experiences and perceptions of work capacity and how it can be assessed.	Individual interviews 2007–2008 Qualitative content analysis	14 physicians from different specialties (6 primary health care, 3 occupational health, 3 orthopaedic surgery, 2 rehabilitation) Purposive sampling	Assessing work capacity was seen as something vague. Physicians mainly relied on the patient’s story when assessing work capacity. Mutual trust was seen as necessary for a successful assessment. Participants agreed that non-medical factors affect work capacity but disagreed on whether they could be included in the assessment.
Sturesson et al. [35] Sweden	Explore physician and occupational therapist views on work capacity and experiences of work capacity assessments.	Focus groups 2008 Qualitative content analysis	14 physicians (9 general practitioners and 5 physicians at the Swedish social insurance agency) and 23 occupational therapists from primary health care and rehabilitation -	Physicians described work capacity as a complex phenomenon affected by many interacting factors and unique for every individual. They expressed difficulties assessing work ability due to lack of instruments, and did not fully agree on which factors should be included in the assessment.
Wynne-Jones et al. [36] UK	Explore general practitioners’ and physiotherapists’ perceptions of sickness certification in patients with back pain problems.	Semi-structured telephone interviews - Thematic analysis (using constant comparative method)	11 general practitioners and 6 physiotherapists Random sample from respondents to another study who consented to further contact (general practitioners) and snowball sampling (physiotherapists).	The general practitioners rarely initiated discussions about work problems and when they did, they rarely used structured measures. Due to lack of training and skills in occupational health and limited knowledge of the work place physicians felt ill-equipped to offer practical advice and were unsure whether sick leave was in the patient’s best interest.

**Fig. 3:**
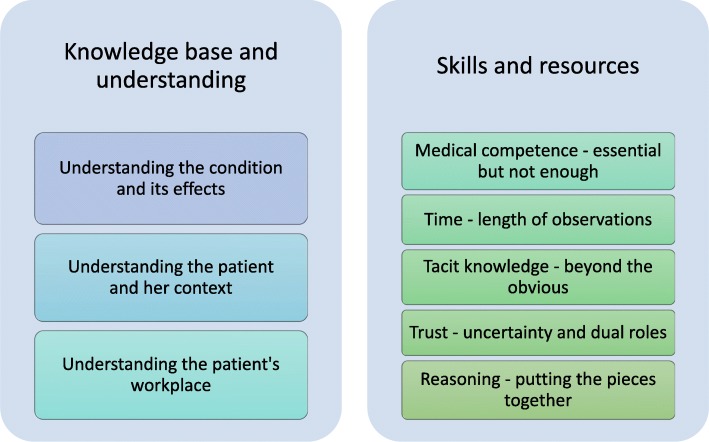
Themes and corresponding categories

As a result of the thematic analysis, two themes were formed: *Knowledge base and understanding* and *Skills and resources*, see Fig. 3. Each is presented below, with corresponding categories. Quotations from the primary studies are added for illustration, within single quotation marks when quoting participants and without quotation marks when quoting the authors.

### Knowledge base and understanding

This theme concerns what information physicians seek in the assessment. Overall, we found that physicians’ information seeking process revolve around three areas: the condition, the patient and the patient’s workplace, and that the focus and depth of the inquiry about these may differ.

#### Understanding the condition and its effects

Physicians look for symptoms, signs and what they describe as “objective” findings, such as lab tests or radiography, to establish diagnosis, severity and prognosis [25, 26, 32]. Then, they seek information about functional limitations related to the diagnosis, such as disturbed sleep, decreased ability to walk or concentration difficulties [26, 27, 34]. Lastly, they try to understand the effects of the functional limitations on daily life and work, e.g. whether the patient still can walk the dog, take part in social life or perform work tasks [27, 31, 34, 35].

Participants in some studies stated that the diagnosis is actually not central in the assessment [27, 31, 35]. However, it is necessary for eligibility and therefore, lack of “objective” findings was widely recognized as problematic as it caused diagnostic uncertainty and problems with verification of the complaints [26, 28, 32, 34, 35]. Subjective complaints, such as pain or tiredness, were found difficult to assess and often perceived as associated with more complex problems [26, 28, 32].

We also found that physicians considered different aspects depending on the diagnosis: for physical conditions there was a focus on investigating and verifying loss of physical functioning while for psychiatric conditions medical evidence was sought after to a lesser extent and questions focused more on social aspects of private and working life [25, 31]. Furthermore, physician affiliation affected the extent of the information seeking [30, 34].

Functioning was described as a multidimensional phenomenon involving physical, mental and social ability [26]. Physical functioning was considered the easiest and most commonly assessed dimension [26, 28, 30]. When examining the effects of the functional limitations, the focus was sometimes more on how the condition affected private life rather than work life [27, 31, 34].

*Quite often doctors use a so-called ‘day-story’ […]. These descriptions make clear what patients are still able to do and what they cannot do as a result of their illness or disability. A ‘day-story’ thus provides clues as to what tasks the client may be able to perform at work.* (Meershoek et al. (2007), p.502).

#### Understanding the patient and her context

Besides the present condition, physicians wanted to know the patient’s previous medical history and history of sick leave [25, 27]. Furthermore, the patient’s social situation was an area of concern [27, 28, 32]. A recurrent finding was the importance of having a holistic approach. Medical and non-medical factors were seen as inseparable and equally important as they interact and affect each other; therefore, understanding both the patient and her context was considered vital to assess the patient’s work capacity [26, 27, 32, 33, 35]. Family situation, conflicts in relationships, social life, financial worries, addiction and lack of social support were all considered important factors for work ability [25, 27, 28, 32, 35]. Personal history and competences were also of interest, e.g. upbringing, education and work history [27, 35]. Attitudes such as self-image, fears, motivation to return to work and the patient’s opinion of own work ability were considered vital [27, 28, 31, 34, 35]. Also, the patient’s efforts to get better and to return to work were considered [27, 31].

The impact of psychosocial factors was seen as adding complexity to the assessment [26, 28, 32]. So was the uniqueness of every patient, calling for individualized assessments [35]. Furthermore, we found uncertainties among physicians as to which non-medical factors could actually be included in the assessment [27, 33–35].

*‘yes, family roles, family, interplay and how this person sees himself, his life and his ability*. *.*. *And that’s the point of departure, I think, if you want to support healing or influence work ability, you need to look at more than work ability in relation to ordinary work demands.’* (Stigmar et al. (2010), p. 1784).

#### Understanding the patient’s work place

The physicians acknowledged that understanding the work place was necessary when assessing work capacity. They were aware of both physical and social aspects of the work place that could affect the ability to work, but stressed the difficulties in assessing them [26, 28, 32, 33, 35]. The main source of information regarding the work place was the patient, and the inquiry was narrow and unstructured [31, 35, 36]. Contact with the employer was rare, as was work place visits [28]. Physicians expressed that the limited knowledge of the work place caused feelings of uncertainty regarding the assessment [26, 28, 29].

Questions about work tasks and demands could include aspects such as heavy lifting, opportunity to take a break or adjust work pace [25, 35]. Questions about the social situation at work could include asking about support mechanisms, conflicts, bullying, work culture/policies and job satisfaction [25, 27, 35]. Also, the employer’s attitude and actions were mentioned as important questions [27, 28, 35, 36]. One study in particular showed that for physical conditions there was a focus on the physical aspects of work while for psychiatric conditions there was a stronger focus on social issues [25].

*‘It’s not like we stand around and load the patient up with weights to see if he can handle 3 kilos? [ …*] *And ok, he could choose to lift half of that – but then he’ll be working too slowly! These things … are impossible for us to assess.’* (Krohne & Brage (2008), p. 854).

### Skills and resources

This theme refers to what skills, methods and resources physicians use to obtain and evaluate the information in the assessment. Overall, they use a wide variety of skills, medical competence being only one of them.

#### Medical competence – essential but not enough

Medical competence was seen as essential; a good medical examination and investigation was said to be the foundation of the assessment [34]. The patient’s story was the main source of information. Clinical diagnostics such as examinations, clinical tests, lab tests, observation and rating scales were used, more in the case of physical conditions than psychiatric [25–27]. Comparing and looking for patterns was mentioned [27, 29]. Referral could be used in cases of uncertainty; physiotherapists or occupational therapists were mentioned as a possible help [26, 28, 32, 35]. Besides that, collaboration with other stakeholders, such as health care and employers, was seen as desirable but difficult to achieve [28, 32, 34].

Though fundamental, we found that the medical competence was not enough for the assessment of work capacity [27, 28, 34]. Work capacity was described as multidimensional and dynamic: many complex factors interact, the same condition affects individuals differently depending on their unique situation, stakeholder actions can affect work capacity in negative or positive ways and what is considered as “being able to work” is influenced by politics, media and the labour market [27, 33–35]. Physicians felt ill-equipped to handle this complexity [26, 28, 32, 34] and expressed a lack of skills in occupational health and insurance medicine [32, 34, 36]. Also, instruments to assess work ability in an objective way were lacking [29, 32, 34].

*Medical knowledge is incorporated into the process of evaluation, not as a decisive factor, but to help determine whether a client is exaggerating his or her complaints. It serves as a point of reference in an evaluation. But it cannot fully account for doctors’ evaluation of clients’ capacities to work, and does not even serve as the basis of their decision in any substantive sense.* (Meershoek et al. (2007), p. 509).

*‘So it is definitely not only diagnostic, it is much more. The sooner I know about [the entire problem] the safer I feel, even if this person is still on sick leave, that I know that there is nothing else that I missed at the beginning.’* (Sturesson et al. (2013), p. 122).

#### Time – length of observations

Time was mentioned as an important tool as duration of time together with the patient determined the physicians’ possibilities to capture adequate information [35]. Repeated assessments with time in between enabled the physician to see a process/development which further informed their decision [29]. However, lack of time in daily work often meant short and irregular consultations with the patient where the time-consuming assessment and certification of work capacity was hampered and/or not prioritized, limiting the possibilities to make proper assessments [28, 33, 34].

#### Tacit knowledge – beyond the obvious

Expressions like *intuition, gut feeling, impression* and *feeling of* occurred in several studies, i.e. expressions that could be understood as referencing tacit knowledge [27–29, 34]. One study explicitly stated that the physicians found it difficult to describe the assessment and referred to it as taking place “in the back of our minds” (Krohne & Brage (2007), p. 173) [29]. Physicians also stated that they could intuitively sense when something went wrong or if there was risk of long-term sick leave [28, 34].

*‘So this is in the gut – much more than measuring the bending angle of … ’* (Krohne & Brage (2007), p. 174).

#### Trust – uncertainty and dual roles

A recurrent finding was the issue of subjectivity and uncertainty in work ability assessments [26–29, 32, 34]. Therefore, according to many informants, listening to and trusting the patient’s story was essential and constituted the basis of the assessment [28, 32, 34, 35]. Trust was seen as a necessity for a good consultation, and a way to handle the fact that it is impossible to prove many (or most) cases as well as knowing if the patient is lying or not [27, 32]. Other participants, however, were not comfortable with this [26, 28, 29] and described it as guessing and “compromising with one’s conscience as gatekeeper” (Krohne & Brage (2007), p. 175). The level of trust was affected by several factors, such as previous knowledge of the patient, the way the patient described and handled the situation and the physician’s own workload [27–29].

*‘I never know if someone is lying to me or not lying to me. I never know if my assessment is true or not true, and I never know how to put people back to work in an appropriate time. I don’t know if I should be pushing them to go back to work sooner or being slow and protracted; so I am really relying on the patient’s word rather than anything else [ …*]’ (Soklaridis et al. (2011), p. 206).

*‘If they have an injury or a disease then there is, of course, no problem. It’s those who complain of being tired, having pain somewhere, or do not have the strength who are the hard ones, those with vague symptoms. And you don’t really know what they work with or who their colleagues are, and you don’t have the time to visit the workplace to see how it really works and how hard it is. It is only guesses from my point of view, you guess all the time, and that does not feel good.’* (Nilsing et al. (2013), p. 455).

#### Reasoning – putting the pieces together

Related to intuition and trust is the aspect of *reasoning*. Due to the complexity of the matter, the assessment was based on an argumentation rather than a measurement. In most cases, work ability cannot be measured or proven with absolute objectivity. Instead, the physicians reason their way to a probable, fair enough, assessment [27, 31, 34]. They relate and weigh available information to assess the plausibility of the patient’s complaints – is it reasonable? Does it make sense? Finally, possible consequences of the decision are taken in to account, including the risk of medicalisation, the risk of aggravating the condition and the risk of the patient taking on a sick role if he/she is denied sick leave [27, 34].

‘*Actually as a physician I judge whether the patient’s own appraisal is reasonable or not.’* (Stigmar et al. (2010), p. 1784).

*‘Well, in fact it is better to consider the relation between resources or capacities and demands than the diagnosis. People always have complaints, whether these complaints are covered by a diagnosis is in fact not that relevant for a social medical judgement. Sometimes it is, but usually it isn’t, the precise diagnosis. It is more important to become convinced that the complaints are plausible, very often you cannot prove them, that’s also useless to do, because then you start a process of medicalisation.’* (Meershoek et al. (2007), p. 501).

## Discussion

Although a central part of the sickness absence and rehabilitation process, physicians’ work with assessing work capacity has received little attention. Previous studies have examined physicians’ sickness certification practices both quantitatively and qualitatively regarding frequency [18], experiences and feelings [38–40], perceived problems [41, 42] and strategies [43] but their actual clinical practice when determining work capacity has received little attention. This is, to our knowledge, the first synthesis of qualitative research on the matter.

The synthesis shows that physicians, in the work capacity assessment, seek to form a knowledge base including understanding the condition, the patient and the patient’s workplace. They recognise the importance of both medical and non-medical factors and, in accordance, use both medical and non-medical skills and resources to acquire and evaluate the information.

Overall, we found that the physicians had a holistic view on work capacity, considering information about medical, personal and contextual issues. This approach is in line with the International Classification of Functioning, Disability and Health (ICF), a biopsychosocial model of disability, where a person’s level of functioning is seen as “a dynamic interaction between her or his health condition, environmental factors, and personal factors” [44].

However, we found differences among the physicians regarding which aspects were emphasised. This could be related to institutional factors such as type of compensation system [45], but also to individual factors such as level of experience and knowledge or personal attitudes towards sick leave and return to work [46]. In our study, the differences were seen both across and within studies indicating individual rather than institutional differences. It is striking how little the medical part was discussed. Even though a thorough medical assessment was considered essential for a good assessment and understanding of the patient’s work capacity, many considered the diagnosis peripheral and instead emphasised the social evaluation. Work capacity was described as being personal – individual factors had to be known. Within the medical sphere, physical conditions and physical aspects of functioning were considered the easiest to assess and were associated with less focus on social issues. That physicians seek different kinds of information depending on diagnosis might be due to their empirical knowledge about what is usually relevant for a given condition but it could also reflect the tensions between the different types of knowledge physicians consider in these evaluations – the “objective” (possible to observe or measure) and the “subjective” (descriptions, the lived experience of the condition) – and how they can be assessed. Understanding the wider and individual aspects of illness requires more time than is usually available. Lack of time therefore means that these aspects must be omitted or reduced to a minimum of attention. This explains some of the frustration, and in some cases resignation, expressed – they know what must be known for a complete assessment, but do not have the resources to explore it.

To appreciate work capacity, functioning must be compared to actual work demands [47]. Still, our findings suggest that physicians’ enquiry about work is limited. Again, lack of resources is a concern. As previously shown, general practitioners often have limited training in insurance medicine and occupational health [19, 48], which impedes their ability to understand how different work place factors affect work capacity. Lack of time and methods to assess the work place were other important reasons reported in the articles. Thus, physicians’ focus on functioning in private life may be deliberate: Meershoek et al. (2007) describe that the day-story “provides clues as to what tasks the client may be able to perform at work” (Meershoek et al. 2007, p. 503), and as such is a relevant enquiry in the work capacity assessment. Similarly, in a qualitative study where health professionals described their understanding of work capacity in patients with depression and anxiety, “outside work” incapacities were considered part of the capacity to work, exemplified by descriptions of how patients used all their energy at work while life outside work was falling apart [49]. This was perceived as conflicting with the medico-legal perspective where the basis of the assessment is limited to work only. We found similar discussions about psychosocial aspects; while these were often described as most important for the ability to work, we found in some articles that the physicians expressed an uncertainty as to if and how to include them in the assessment, and some physicians explicitly did not.

While medical competence was seen as an important basis for the work capacity assessment, other competences such as intuition, trust and reasoning were identified as necessary to assess the authenticity of the patient’s claims and translate the findings into a final assessment. All of these skills are to some extent part of a physician’s ordinary toolbox. But while in a medical assessment there are techniques and measurements to support the decision, in the work capacity assessment such resources are scarce and the non-medical skills play a far greater role.

Clinical reasoning can in its simplest form be seen as a technical matter, a way of solving problems with definite solutions, like solving a puzzle. For the physician, this would mean finding the diagnosis and suitable means to treat it. It can, however, be argued that clinical reasoning is much more than that. According to Mattingly, it means figuring out how to best deal with the particular situation which involves conscious and unconscious assessing and reassessing “en route” [50]. We interpreted the reasoning going on in the work capacity assessment as the latter; a deliberating, considering aspects beyond the obvious. This type of reasoning puts the theory in context, applies it to the specific situation of the individual case. This correlates well with the finding of Bertilsson et al. (2018) [45] that in the “work capacity puzzle”, the physicians both create and fit the pieces; a process requiring both tacit and explicit knowledge. Similarly, a study comparing work capacity assessments of patients with severe subjective health complaints by physicians in five European countries showed significant differences among physicians but also unexpected similarities, suggesting that “physicians share tacit knowledge regarding sick-leave decision making” for this patient group [51]. Theoretical and empirical knowledge is used jointly to assess whether the patient’s complaints are reasonable, and how to deal with them. In this process, intuition and trust also play a role. According to a study by Mårtensson & Hensing (2012) [52] a trustful relationship was identified as important for informed decision making. Their findings concerned patients but support our results that the same is true for physicians – trusting the patient facilitates their decision making.

In contrast to the use of non-medical skills is the call for “objective” ways to measure work capacity. Increasingly, instruments and guidelines are being used in health care. As a complement to the consultation they can increase diagnostic sensitivity, but there is also a risk of overreliance on their results [53]. Instruments are by nature generalist and static, designed to capture common features – they cannot detect subtle changes, relate to previous assessments or reassess “en route”. Furthermore, given the limited evidence concerning how work capacity is affected by specific health conditions and factors at work, and how to best promote return to work [54], the evidence base for instruments for work capacity assessments is rather weak. Knowledge about the shortcomings of a test, and when to use it, is essential to make correct interpretations of the results; a pathological test result will not necessarily lead to initiating treatment and a normal test result can be ignored if other findings, or the“ gut feeling”, suggest that something is wrong. Within physicians’ clinical expertise lies the ability to put the theories and recommendations into a context, to know the standard procedure but also when to deviate from it. The idea to standardize healthcare has been widely accepted and implemented along with the advance of evidence-based medicine (EBM), proposing that aligning health care professionals’ practice reduces unwanted variation and error. But critics have put forward that an excessive “one size fits all” approach comes at the expense of professional performance and ultimately patient care, as the “art of medicine” is neglected and loyalty towards the patient is replaced by a loyalty to the system [55]. Our results suggest that a fully standardized assessment of work capacity is not achievable, or even desirable. The physicians emphasize the uniqueness in every assessment; there is a dimension that arise there and then, in the meeting between doctor and patient, that is beyond measurements and protocols. Its dynamic nature prevents it from being fully captured in static templates. The assessment is contextual, even on a macro level [56]. In a focus group study, insurance physicians assessing disability benefits for cancer survivors expressed that they considered the standardized forms to lack important aspects and being “not well suited to monitor a RTW trajectory” (which we interpret as being too static). The same participants also discussed guidelines, and while some physicians reported that the guidelines provided a starting point for discussion and supported their decision, others were more negative, stating that the guidelines were too general and “did not support their professional judgement in translating gathered data (i.e. information provided by GP, consultant, occupational health service and cancer survivor) into functional abilities” [57]. In Sweden, a decision aid for sickness certification was introduced in 2007–2008 to align physicians’ sick-listing practices. It has been reported to facilitate physicians’ communication with patients regarding sick leave (e.g. as a support for denying sick leave) [58], but has been criticised for its format and content, restricting what aspects of their knowledge the physicians can communicate to the authorities [59]. Similarly, Aarseth et al. (2017) [60] analysed the sickness certificate used in Norway and concluded that it has a strong focus on disease and do not ask for personal aspects such as the social context, leading to valuable information being omitted. Again, the tension between the “objective” and the “subjective” is evident. As pointed out in included articles, physicians have to conform to rules and predefined criteria that do not always harmonize with their view of the matter. This, according to Meershoek et al. [27], covers up their line of reasoning, leading to less well-founded decisions about the patient’s eligibility for benefits.

Considering the complexity of the assessment of work capacity, perhaps the whole notion of what an “objective” evaluation means needs to be reconsidered. It calls for a different view on objectivity – *contextual objectivity* rather than *absolute objectivity*. The idea that absolute objectivity is possible is simplistic and implies that it is possible to separate the assessment from the many integrated factors that together compose the patient’s life and work capacity. In contrast, the concept of contextual objectivity recognizes that the assessment is a reflection of the unique composition of factors that make up the specific situation of the patient at that specific moment.

In the assessment of work capacity, physicians rely on their non-medical skills to a higher degree than in ordinary clinical work. These skills are highly relevant but need to be complemented with access to appropriate resources. To determine if the reduced function is due to the patient’s condition, if and how it affects the patient’s capacity to work and how it all relates to the overall aim to improve the patient’s health, both clinical and work place expertise and reasoning is needed. To achieve this, we believe that certifying physicians need more formal training in insurance medicine to understand the intricate associations between health, work and social security (not applicable to insurance physicians in the Netherlands who receive 4 years of training in this). With this knowledge at hand, and sufficient resources such as adequate amounts of time, physicians would be better equipped to evaluate the work capacity in greater depth.

In the updated search, one article was found eligible for inclusion [37]. This large qualitative study explored how physicians assess work capacity in patients with common mental disorders, and their findings support our results. They highlight the complexity of the assessment and stress the importance of a comprehensive understanding of the condition, patient and work place as well as the use of time, tacit knowledge and reasoning for an adequate assessment.

### Methodological considerations

As we were aiming to identify all relevant articles on the topic but expected this number to be small, we chose a systematic and comprehensive search strategy resulting in many hits. However, for a number of reasons, our review is likely not exhaustive. Due to unsatisfactory indexing, searching systematically for qualitative articles is difficult [61]. The same is true for articles within sickness absence research. Furthermore, including additional search terms and other languages than English might have increased the number of relevant articles.

The analysis was at large carried out by the first author, a medical doctor with 2 years’ experience of working with sickness certification in Swedish primary health care. Her understanding of the situation was an advantage in the analysis process as it facilitated identification and categorization of relevant data. At the same time, preconceptions could have influenced the way data was interpreted. To reduce this risk, data was checked against the context it originated from and findings were discussed continuously with co-authors during the analysis process.

Included studies varied regarding setting and sample. Different countries have different sick leave regulations and health care systems which influence the physician’s role and practices. For example, in the Netherlands, specialized insurance physicians work only with sickness certification, while in most other countries GPs issue sickness certificates. Recently, in the UK, the role of the GP has been reduced and the assessment is instead made by a civil servant [56]. These contexts were rarely described or discussed in included articles, and we were therefore unable to fully examine them and their effect on the assessment. A more thorough analysis of how differences in welfare and health care systems influence the assessment of work capacity would be an interesting approach for further research.

Included articles contributed to a varying degree to our results, mainly due to their richness of data in relation to our research question; depending on their aim and method, some presented a comprehensive picture of the assessment while other gave a more demarcated description. All included articles were considered to be of acceptable quality, but we identified some common shortcomings that might have affected our results in terms of validity and transferability. The authors often failed to explicitly describe the relationship between researcher and informants and to discuss the importance of this for the performance of the study. To a very small extent the researcher’s own knowledge, experience and preconceptions were discussed in relation to the analysis of data. Also, theoretical frameworks were often lacking. Nevertheless, we believe that the included material gives a sufficiently broad overview of the practices of physicians and that our findings can contribute to a more comprehensive understanding of the aspects that need to be considered in the assessment of work capacity. In addition, the study can serve the purpose of highlighting areas of further and more in-depth research.

## Conclusion

A good assessment of work capacity is the basis of a good certification and rehabilitation process, which will gain patients, physicians and society as a whole. But what is good? And how do we achieve it?

It seems that physicians’ own competences and skills go quite a long way in the work capacity assessment. We found, however, that many physicians lack proper resources. Time constraints, poor collaboration and insufficient training in insurance medicine hamper their possibilities to make good assessments. Also, the limited knowledge of work place factors makes the translation of functioning into work capacity difficult. Hence, the problem is not primarily a lack of instruments and validated methods but rather a lack of basic prerequisites. Providing physicians with these will most likely benefit the assessments. Equally important, the notion of an “objective” evaluation needs to be questioned, calling for a greater appreciation of the complexity of the assessment and the role of professional judgement.

## Supplementary information

**Additional file 1.** ENTREQ checklist (Enhancing transparency in reporting the synthesis of qualitative research)

**Additional file 2.** CASP Checklist: 10 questions to help you make sense of a Qualitative research

## Data Availability

All data generated or analysed during this study are included in this published article.

## Abbreviations

GP: General Practitioner
IP: Insurance Physician
ICF: International Classification of Functioning, Disability and Health
PHCC: Primary Health Care Centre

## Footnotes

1 In the Netherlands, these registered medical specialists receive 4 years of training in assessing work capacity and carry out the assessments on a daily basis.
